# Editorial: Mechanisms of Cytokinesis in Eukaryotes

**DOI:** 10.3389/fcell.2021.668705

**Published:** 2021-03-19

**Authors:** Issei Mabuchi, Maria Grazia Giansanti, Fred Chang

**Affiliations:** ^1^Department of Life Sciences, Graduate School of Arts and Sciences, The University of Tokyo, Tokyo, Japan; ^2^Institute of Human Culture Studies, Otsuma Women's University, Tokyo, Japan; ^3^Institute of Molecular Biology and Pathology, Italian National Research Council, Rome, Italy; ^4^Department of Cell and Tissue Biology, University of California, San Francisco, San Francisco, CA, United States

**Keywords:** contractile ring, intercellular bridge, abscission, actin, myosin, Rho, Rac, ESCRT

The study of cytokinesis, the final stages of cell division, is one of the most active fields in cell biology. This field encompasses investigations on a multitude of diverse cellular processes including cytoskeletal regulation, membrane dynamics, signaling, and cell mechanics, all studied using diverse approaches in model organisms ranging from bacteria and archaea to yeast, plants, and animal cells. This series of articles in Frontiers of Cell and Developmental Biology highlights recent process on our understanding of cytokinesis in eukaryotic cells.

Many eukaryotic cells divide by virtue of constriction of the acto-myosin based contractile ring (CR, Figure 1), which was discovered by Schroeder (1968, 1970) using electron microscopy in jellyfish eggs and HeLa cells. Myosin-II is the essential motor for cytokinesis in animal cells (Mabuchi and Okuno, 1977). In this issue, Wang et al. compare the functions of different myosin IIs in cytokinesis in budding yeast, fission yeast and human cells. Budding yeast myosin-II seems to form filaments at the division site, though *in vitro* assembly has not been reported so far. However, it is curious that MYO1 heads (motor domains) are dispensable for division in budding yeast. In fission yeast, the structure/function of the two myosin-IIs (the essential Myo2 and non-essential Myp2) and their roles in the formation and constriction of the CR have been extensively characterized. Myo2 has not been reported to form filaments. Mammalian cells express three non-muscle myosin-IIs (NM-IIA, IIB, and IIC) which all localize to the division site. They form filaments but NM-IIA filaments have recently been reported to form stacks during furrowing, presumably for effective force production (Fenix et al., 2016). Wang et al. claim that the furrowing has two stages. First, the furrow ingresses until it reaches a diameter of 1.5–2 μm (Wang et al., 2019). This process is driven by all the isoforms. Next, IIB causes further constriction to form the thin intercellular bridge that is resolved later by abscission (see below).

**Figure 1:**
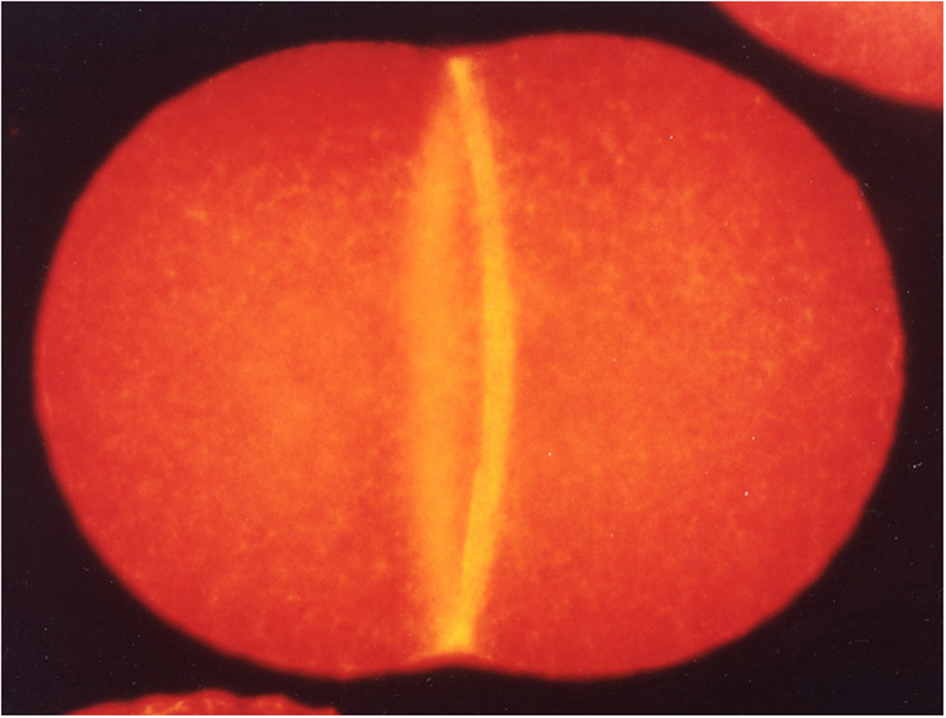
A dividing sea urchin (*Hemicentrotus pulcherrimus*) egg fluorescently stained for F-actin to visualize the CR (Mabuchi, 2008).

A key regulator for cytokinesis is the Rho GTPase, the first regulatory protein factor identified for CR formation (Kishi et al., 1993; Mabuchi et al., 1993). Carim et al. describe roles of two Rho-dependent cytokinetic networks, actin-myosin and anillin-septin networks, both of which are independently anchored to the plasma membrane. They describe a scenario of Rho-dependent formation and contraction of the CR. Specifically, they propose that, during closure of the ring at the point at which its tension reaches a threshold via ring contraction, a Rho-dependent elimination of the membrane microdomains anchored by anillin and septin occurs.

The Rac GTPase, another Rho family member, also contributes to cytokinesis. Pal et al. manipulate the activities of Rac and its effector Arp2/3 during mitotic divisions of early sea urchin embryos, where these factors are dispensable for cytokinesis, as well as in meiotic divisions of sea star oocytes that depend on these proteins for polar body extrusion. In both cases, expression of activated Rac produces an inhibitory effect on cytokinesis that is suppressed by an effector binding mutation or direct inhibition of Arp2/3. These results indicate that the branched actin networks nucleated by Arp2/3 may act as a brake against Rho-dependent contractility at the cleavage site.

A number of mechanisms have been shown to drive contractile ring assembly in different cell types and contexts. In *C. elegans* embryos, cortical flow has been considered to be the main driver of CR formation (Reinann et al., 2016). However, a research article by Leite et al. provides evidence that this is not the case. Instead, they show that the actin filament cross-linker plastin plays a role in the assembly of the ring along with myosin-II.

Although a core set of conserved cytokinesis proteins has been extensively studied (Balasubramanian et al., 2004; Pollard and O'Shaughnessy, 2019), there are still new factors to be found. Nguyen and Robinson review recent discoveries showing proteins that have been implicated previously in other cellular functions also have unexpected roles in cytokinesis. These new cytokinesis players named “unusual suspects” include membrane associated proteins such as discoidin and intracellular chloride channels, RNA-related proteins, the nuclear proteins Importin β, Ran and Lamin B and metabolic enzymes. Investigation of these novel cytokinesis proteins will reveal new cross-talk and network integration and help complete the puzzle of this intricate process.

Successful cytokinesis involves a substantial increase of total surface area, which depends on membrane trafficking from internal stores. However, because of complex geometry of the plasma membrane in animal cells, this increase has been difficult to measure accurately. To eliminate the contribution of small surface membrane reservoirs, Tanaka et al. measure the total increase of surface area in dividing *Dictyostelium* cells flattened by an agar overlay method. Under these conditions, the total surface area increases during cytokinesis by about 20% through an imbalance between exocytosis and endocytosis. In contrast with previous reports, although clathrin-dependent endocytosis was suppressed during cell division, it did not contribute significantly to the changes of cell surface area.

Following the constriction of the CR, the final stage in animal cell cytokinesis is abscission, the process of physical separation of the daughter cells. This step, which can occur significantly after CR constriction, involves the scission of a midbody structure that is densely populated with microtubules and proteins involved in membrane trafficking. The endosomal sorting complex required for transport (ESCRT) pathway is a key factor for this abscission process (Carton and Martin-Serrano, 2007; Morita et al., 2007; Hurley and Hanson, 2010). Horváth and Müller-Reichert review the role of ESCRT in abscission from a structural point of view. Cryo-tomography of the midzone structure reveals the assembly of ESCRTs into remarkable spiral structures surrounding the dense midbody structure. Structural studies provide new insights into the molecular bases for their assembly and regulation of membrane curvature. This review discusses current models for how ESCRT ultimately contributes to membrane deformation and fusion to resolve the intercellular bridge structures.

The ESCRT machinery represents a primordial cytokinetic mechanism that pre-dates the actin-based contractile ring in the evolution of eukaryotes (Lindås et al., 2008; Samson et al., 2008). It mediates cytokinesis in some archaea and is likely to mediate cytokinesis in much of the eukaryotic tree. To test another branch of the tree, a research article from Yagisawa et al. reveals a role for ESCRT in the red algae *C. morelae*, which divides by furrow constriction but without myosin or septins. They show that ESCRT resides at the intercellular bridge, and provide initial evidence that it participates in abscission in this algal cell.

After abscission, the midbody in animal cells can persist as a midbody remnant in the next generation, where it has been shown to have post-mitotic functions such as polarity regulation and stem cell differentiation. This intriguing remnant structure may be internalized to function inside the cell, be released, or be degraded through autophagy. In a research article, Sardina et al. identify a new regulator of midbody remnant, a protein kinase HIPK2 that promotes the removal of the midbody remnant through autophagy.

In summary, this set of articles and reviews illustrates the impressive breadth of the current cytokinesis field in the investigations of the diverse molecular processes responsible for this essential process of cell division.

## Author Contributions

All authors contributed to both writing and editing the draft.

## Conflict of Interest

The authors declare that the research was conducted in the absence of any commercial or financial relationships that could be construed as a potential conflict of interest.
